# Entomopathogenic fungus *Beauveria bassiana*–based bioinsecticide suppresses severity of powdery mildews of vegetables by inducing the plant defense responses

**DOI:** 10.3389/fpls.2023.1211825

**Published:** 2023-08-24

**Authors:** Yuichiro Iida, Yumiko Higashi, Oumi Nishi, Mariko Kouda, Kazuya Maeda, Kandai Yoshida, Shunsuke Asano, Taku Kawakami, Kaori Nakajima, Katsutoshi Kuroda, Chiharu Tanaka, Ayano Sasaki, Katsumi Kamiya, Naho Yamagishi, Masashi Fujinaga, Fumihiro Terami, Satoshi Yamanaka, Masaharu Kubota

**Affiliations:** ^1^ Laboratory of Plant Pathology, Faculty of Agriculture, Setsunan University, Hirakata, Japan; ^2^ National Agriculture and Food Research Organization, Tsu, Japan; ^3^ Nara Prefecture Agricultural Research and Development Center, Sakurai, Japan; ^4^ Mie Prefecture Agricultural Research Institute, Matsusaka, Japan; ^5^ Gifu Prefectural Agricultural Technology Center, Gifu, Japan; ^6^ Nagano Vegetable and Ornamental Crops Experiment Station, Shiojiri, Japan; ^7^ Arysta LifeScience Corporation, Tokyo, Japan; ^8^ National Agriculture and Food Research Organization, Tsukuba, Japan

**Keywords:** endophyte, biofungicide, dual control, biocontrol agent, salicylic acid, induced resistance, plant-microbe interaction, *Podosphaera xanthii*

## Abstract

The entomopathogenic fungus *Beauveria bassiana* is used commercially as a microbial insecticides against a wide range of agricultural insect pests. Some strains of *B. bassiana* protect the plants from pathogens, but the underlying mechanisms are largely unknown. Here, we found that prophylactic sprays of commercial bioinsecticide Botanigard on cucumber, tomato, and strawberry plants suppressed the severity of economically damaging powdery mildews. On leaf surfaces, hyphal elongation and spore germination of cucumber powdery mildew, *Podosphaera xanthii*, were inhibited, but *B. bassiana* strain GHA, the active ingredient isolated from Botanigard, only inhibited hyphal elongation but had no effect on spore germination of *P. xanthii*. In addition, strain GHA suppressed powdery mildew symptoms locally, not systemically. Treatment with Botanigard and strain GHA induced a hypersensitive response (HR)–like cell death in epidermal cells of the cucumber leaves in a concentration-dependent manner and inhibited penetration by *P. xanthii*. Transcriptome analysis and mass spectrometry revealed that GHA induced expression of salicylic acid (SA)–related genes, and treatment with Botanigard and GHA increased the SA level in the cucumber leaves. In *NahG*-transgenic tomato plants, which do not accumulate SA, the biocontrol effect of tomato powdery mildew by GHA was significantly reduced. These results suggested that *B. bassiana* GHA induces SA accumulation, leading to the induction of HR-like cell death against powdery mildew and subsequent suppression of fungal penetration. Thus, Botanigard has the potential to control both insect pests and plant diseases.

## Introduction

The entomopathogenic filamentous fungus *Beauveria bassiana* (Balsamo-Crivelli) Vuillemin has a wide range of arthropod insect hosts and is common from arctic to tropical regions throughout the world (Feng et al., 1994). Its spores attach to the body of the insect, germinate, and form appressoria. Then, hyphae secrete hydrolytic enzymes such as proteases, lipases, and chitinases to penetrate the insect’s cuticle and then to colonize the nutrient-rich hemolymph (Feng et al., 1994). Once the host insects die, *B. bassiana* sporulates on the surface of the insect, from where the aerial spores are dispersed. This cosmopolitan and naturally soil-inhabiting fungus infects agriculturally important pests such as thrips, whitefly, spider mites, and aphids that have developed a high resistance to chemical insecticides and have become a major issue (Kliot et al., 2016). *B. bassiana* is used in a variety of agricultural situations to manage a diversity of pests, with few published non-target effects (Zimmermann, 2007; Mascarin and Jaronski, 2016). Thus, this entomopathogen is now used as an active ingredient in commercial microbial insecticides and widely used as a sustainable biocontrol agent (Sullivan et al., 2022).

In addition, certain strains of *B. bassiana* can prevent plant diseases. Hence, *B. bassiana* might exert a dual biocontrol effect against both pathogens and insect pests of plants (Ownley et al., 2008; Quesada-Moraga et al., 2009). Some *B. bassiana* strains are antagonistic to fungal pathogens, soil-borne *Rhizoctonia solani* and *Gaeumannomyces graminis* var. *tritici*, and air-borne *Botrytis cinerea* and *Alternaria alternata*, but no modes of action have been mentioned (Renwick et al., 1991; Ownley et al., 2008; Sinno et al., 2021). Strains BG11 and FRh2 reduce the incidence and severity of symptoms caused by *Sclerotinia sclerotiorum* and alter the expression of genes related to plant defense, phytohormones, and secondary metabolites in a strain-specific manner, but the amounts of phytohormones and secondary metabolite do not change (Raad et al., 2019). Phytohormones play a central role in the regulation of systemically induced plant resistance. Some of non-pathogenic beneficial microorganisms living in the rhizosphere trigger induced systemic resistance via jasmonic acid (JA) and ethylene (ET) signaling pathway against pathogens (Sun and Zhang, 2021). In contrast, salicylic acid (SA) plays a central role in systemic acquired resistance (SAR) induced by necrotizing pathogen (Sun and Zhang, 2021). Little is known as to how phytohormones are involved in the mechanisms by which *B. bassiana* prevents plant diseases caused by pathogens so far.

*B. bassiana* strains have been found to live as epiphytes and as endophytes in plant tissues without causing any symptoms in a very wide variety of plants, monocots (maize, sorghum, and orchard grass), eudicots (cucumber, tomato, and banana), and woody plants (cacao, date palm, and coffee) (Posada and Vega, 2005; Quesada-Moraga et al., 2014; Klieber and Reineke, 2016; Jaber and Ownley, 2018; Nishi et al., 2020). In the absence of host insects, *B. bassiana* is likely to live with plants rather than in the soil, based on compatible interactions with plants. Whether *B. bassiana* grows epiphytically or endophytically on a plant depends on the combination of *B. bassiana* strain and plant species. *B. bassiana* ATCC74040 grows well epiphytically and forms spores on the leaf surface of *Vicia faba*, *Brassica napus*, and *Zea mays* (Koch, 2018), whereas strains EABb04/01 and ARSEF3113 are endophytes that enter the leaves through stomata or penetrates an intact epidermis, respectively (Wagner and Lewis, 2000; Quesada-Moraga et al., 2006; Barta, 2018). However, unlike on insects, none of these *B. bassiana* strains form an appressorium to penetrate the plant.

Some strains of *B. bassiana* are beneficial in promoting plant growth or having the potential to translocate nitrogen. Inoculation of *Arabidopsis thaliana* roots with BG11 significantly increased root biomass and number of leaves (Raad et al., 2019). Bb-13 is a plant growth–promoting fungus (PGPF) and enhances root and leaf length and increases plant height and mass (Liu et al., 2022). Hyphae of the entomopathogenic fungus *Metarhizium robertsii* can act as a nitrogen pipeline from insect carcasses to plant roots (Behie et al., 2012), and *B. bassiana* strain 252 has the same capacity, albeit a weak one (Behie and Bidochka, 2014). This nitrogen transfer between entomopathogenic fungi and plants indicates the potential for a major role in nitrogen cycling in ecosystems.

Powdery mildew fungi are obligate biotrophic pathogens and economically destructive on vegetable crops. *Podosphaera xanthii* is a causal agent of powdery mildew on cucurbitaceous plants, and more than 28 physiological races have been identified (Haonan et al., 2020; Wang et al., 2023). *Pseudoidium neolycopersici* (formerly known as *Oidium neolycopersici*) and *Podosphaera aphanis*, the causal agents of powdery mildew in tomato and strawberry, respectively, are also of great importance worldwide (Pathak et al., 2020; Heaven et al., 2023). Powdery mildew first appears as small patches of powdery white growth on leaf and stem surfaces. These patches gradually spread over a large area of tissues (Pathak et al., 2020; Wang et al., 2023). Fungicides from multiple chemical groups have been the most effective tool to manage powdery mildew diseases, but these fungi also develop tolerance to some of these fungicides (Vielba-Fernández et al., 2020).

When a commercial bioinsecticide Botanigard including *B. bassiana* strain GHA as the active ingredient was used to control thrips, whitefly, and spider mites on cucumber plants in greenhouses, we noticed that tomato and cucumber remained atypically free of powdery mildews. Because strain GHA can grow epiphytically and endophytically (Nishi et al., 2020), it may have the potential to suppress disease through its interaction with plants. Here, we aimed to test whether GHA does suppress powdery mildews of different vegetables and to analyze its mechanism of action.

## Materials and methods

### Assessment of the biocontrol effect of Botanigard on the cucumber plants inoculated with powdery mildew *P. xanthii* under the laboratory conditions

The bioinsecticide Botanigard (Botanigard^®^ ES: Arysta LifeScience, Tokyo, Japan) was stored at 4°C before use according to the instruction (1-year shelf life). For treatments, Botanigard was diluted with distilled water to the desired test concentrations and allowed to stand at room temperature for 0.5 to 1 h and then used for experiments according to the manufacturer’s instruction. This formulation contains petroleum distillates in the inert ingredients and *B. bassiana* strain GHA as an active ingredient.

Potted seedlings of cucumber (*Cucumis sativus* L. cv. Sharp 1; Saitama Gensyu Ikuseikai, Saitama, Japan) were grown in a growth chamber at 25°C with a 16-h light/8-h dark photoperiod. Inoculum of cucumber powdery mildew pathogen *P. xanthii* strain pxB (Fukino et al., 2008), kindly provided by Dr. Koichiro Shimomura (National Agriculture Research Organization, Japan), was maintained on potted cucumber plants grown under the same conditions. Four to five leaves of 3- to 4-week-old cucumber plants (*N* = 10) were sprayed with a 1,000-fold dilution of Botanigard (1 × 10^7^ spores/mL, ca. 2 mL per plant) from 3 days before to 3 days after plants, and were inoculated with a spore suspension of the *P. xanthii* inoculum (1 × 10^4^ spores/mL, ca. 2 mL per plant) with three independent replications. The upper surface of all leaves of each plant was covered thoroughly with a spray of Botanigard, the inoculum, or distilled water (mock control).

Disease severity of leaves was rated 12 days after inoculation of powdery mildew on a scale of 0–4: 0, no symptoms; 1, less than 5% of leaf area diseased; 2, 5%–24% diseased area; 3, 25%–49% of leaf area diseased; and 4, more than 50% of leaf area diseased based on the recommendations in the Fungicide Evaluation Manual of the Japan Plant Protection Association for field trials (www.jppa.or.jp/test/04.html). The severity ratings for individual leaves were then used to calculate disease severity as 100[(1n1 + 2n2 + 3n3 + 4n4)/4*N*], where *N* = total number of tested leaves and n1 to n4 = number of leaves scored with each respective score (1–4). The mean disease severity on the mock plants was set as 100, and the relative disease severity was calculated on the basis of the mean (± SD) (more than three independent replications).

For microscopic observations of the fungus after treatment, as described above, the cucumber leaves were sprayed with a 1,000-fold dilution of Botanigard, followed with the powdery mildew spore suspension but with only ca. 0.5 mL per leaf. After 3 days, leaves were cut into 1-cm squares and stained with 0.01% aniline blue (w/v) mixed with lactophenol solution (1:1:1:1 lactic acid, TE-saturated neutral phenol, glycerin, and distilled water). The leaves were boiled for a few seconds and then destained with 5% chloral hydrate solution (w/v). At least 200 spores of *P. xanthii* at five sites on each of three leaves (15 sites total) were observed for germination and hyphal growth using a microscope IX73 (Olympus, Tokyo, Japan) equipped with a U-MWU filter (Olympus). The percentage of spore germination and hyphal growth was calculated on the basis of the area of stained hyphae in 50 mm^2^ using ImageJ version 1.51 (imagej.nih.gov/ij).

### Assessment of the biocontrol effect of Botanigard on the vegetables inoculated with powdery mildews or whitefly in the greenhouse and field

Cucumber (*C. sativus*), melon (*Cucumis melo* L.), tomato (*Solanum lycopersicum* L.), and strawberry (*Fragaria* × *ananassa* Duch.) were grown in the greenhouse, and eggplant (*Solanum melongena* L.) was grown in an open field at different locations as described in **Supplemental Table 1** (*N* = 6 for cucumber, *N* = 3 for tomato and strawberry, and *N* = 2 for melon and eggplant; three independent replicates in each trial). Leaves and stems of 3- to 6-month-old plants of each species above grown in the greenhouse or the field were sprayed with a 1,000-fold dilution of Botanigard 3 to 10 times at about 1-week intervals. Untreated plants served as the control. Plants were inoculated with the powdery mildew species of the respective vegetables (*P. xanthii* for cucumber and melon, *Pseudoidium neolycopersici* for tomato, *Podosphaera aphanis* for strawberry, and *Sphaerotheca fuliginea* for eggplant) either naturally or by planting plants infected with the powdery mildew in the same area. Disease severity was scored 6–13 days after the last application of Botanigard as described above.

In trials using whiteflies, 60–72 female adult whiteflies (*Bemisia tabaci*, B biotype) were released in the greenhouse where 1-month-old tomatoes were growing. Leaves and stems of tomato plants were sprayed with a 1,000-fold dilution of Botanigard 10 times at about 1-week intervals (detailed in **Supplemental Table 1**). The number of young and old larvae and of adult whiteflies on 16 small leaves of four plants was counted with a loupe, and was compared between Botanigard-treated and untreated tomato plants (six independent replicates).

### Inoculation of plants with *B. bassiana* strain GHA

*B. bassiana* strain GHA, the active ingredient in Botanigard, was grown on sabouraud dextrose yeast extract agar (glucose, 20 g; peptone, 2 g; yeast extract, 2 g; agar, 15 g/L) for 2 weeks at 25°C. Conidia were collected in sterile distilled water containing 0.05% (v/v) Tween 20 and filtered through sterile cheesecloth to remove hyphae. The suspensions were washed twice by centrifugation for 5 min at 2,500 × *g*. Concentrations of conidia were determined using a hemocytometer.

We assessed the disease severity of powdery mildew on the leaves treated with GHA. Four to five leaves of 3- to 4-week-old cucumber plants were sprayed with a spore suspension of GHA at 1 × 10^7^, 1 × 10^8^, or 1 × 10^9^ spores/mL, ca. 0.5 mL per leaf, and then with a spore suspension of *P. xanthii* (1 × 10^4^ spores/mL, ca. 0.5 mL per leaf) to cover the upper surface of all leaves as described above. Plants were grown in a growth chamber as described above. Plants treated with distilled water or a 1,000-fold dilution of Botanigard served as the control. Disease severity was assessed 12 days after inoculation of powdery mildew as described above.

At 24 h after inoculation, leaves were stained with 0.01% aniline blue (w/v) mixed with lactophenol solution and destained as described above. The number of hypersensitive response (HR)–like cell death was counted under 100 spores or germ tubes on the stained leaves using a fluorescence microscopy IX73 optical microscope equipped with UV light and a U-MWU filter (Olympus). The percentage of spore germination was calculated for 100 spores.

To clarify whether systemic resistance was induced by *B. bassiana* GHA, one-half of the each detached leaf surface was sprayed with a spore suspension of *B. bassiana* (1 × 10^9^ spores/mL, ca. 0.2 mL per leaf) and the other with water, and, then, the entire leaf was inoculated with a spore suspension of *P. xanthii* (1 × 10^4^ spores/mL, ca. 0.5 mL per leaf). Ten leaves were tested for each treatment. Disease severity relative to that on mock control plants was calculated on the basis of the mean (± SD) for three independent replications as described above.

To elucidate the function of *B. bassiana* GHA as a PGPF, roots of 3- to 4-week-old cucumber plants (*N* = 10) were dipped into a spore suspension of GHA (1 × 10^9^ spores/mL) or sterile distilled water for 30 s and then planted into sterile soil in pots. Above ground parts and root biomass (fresh mass) were weighed after 2 weeks.

All data were analyzed using a Mann–Whitney *U*-test in the program R version 4.0.3 (R Core Team, 2020).

### RNA-seq transcriptome analysis

Cucumber plants were sprayed with a spore suspension of GHA (1 × 10^9^ spores/mL) and then inoculated with a spore suspension of *P. xanthii* (1 × 10^4^ spores/mL) as described above (three independent replications). At 24 h after inoculation, total RNA was extracted from leaves using the NucleoMag RNA kit (MACHEREY-NAGE, Duren, Germany) according to the manufacturer’s instructions. cDNA libraries (150-bp paired-end reads) were prepared using the NEBNext Poly(A) mRNA Magnetic Isolation Module kit (for PolyA selection) and NEB NEXT Directional Ultra RNA Library Prep Kit for Illumina (for strand-specific library) (New England Biolabs, MA, USA) and sequenced using a NovaSeq 6000 platform by Rhelixa (Tokyo, Japan). Sequence data were deposited into the DNA Data Bank of Japan (DDBJ) database (accession PRJDB15569).

Paired-end RNA-seq reads were trimmed using Trimmomatic version 0.38 (Bolger et al., 2014) and then mapped to the cucumber (Chinese Long) v3 genome (cucurbitgenomics.org/organism/20) using HISAT2 version 2.1.0 (Kim et al., 2015). Read counts, fragments per kilobase of exon per million reads, and transcripts per million were calculated using featureCounts version 1.6.3 (Liao et al., 2013). Differentially expressed genes (DEGs) were determined using DESeq2 version 1.24.0 (Anders and Huber, 2010) at *p* <  0.05 after Benjamini–Hochberg adjustment and log_2_|fold change| > 1. The DEGs were then annotated for Gene Ontology (GO) enrichment using TBtools and *p* <  0.05 (Chen et al., 2020a).

### Phytohormone analyses

Cucumber plants sprayed with a spore suspension of GHA and *P. xanthii* were prepared as described above. After 24 h, leaves were cut into 1-cm squares and examined for germination of *P. xanthii* spores as described earlier using a stereomicroscope SZ61 (Olympus). SA and JA were extracted and quantified using liquid chromatography–mass spectrometry as described previously (Seo et al., 2007).

For ethylene (ET) analyses, 10-day-old cucumber seedlings were sprayed with a spore suspension of GHA and powdery mildew as described and planted in soil in 50-mL plastic tubes. After 24 h, a sample was withdrawn from the headspace using a syringe and injected into a gas chromatograph (GC-8A, SHIMADZU, Kyoto, Japan) equipped with an alumina column (Porapak Q 50/80; Shinwa, Kyoto, Japan) and a flame ionization detector (Seo et al., 2007). Data were analyzed using a Mann–Whitney *U*-test.

### Inoculation of *NahG*-transgenic tomato plants with powdery mildew and quantification of pathogen growth

Seedlings of tomato *Solanum lycopersici* L. cv. Moneymaker (MM) and its transgenic line expressing the *NahG* gene (MM-NahG), a bacterial gene encoding salicylate hydroxylase that converts SA to catechol (Brading et al., 2000), which are equally susceptible for powdery mildew of tomato *P. neolycopersici*, were grown in a growth chamber. Seeds of MM-NahG seeds were kindly provided by Professor Tsutomu Arie (Tokyo University of Agriculture and Technology, Japan). Discs (8 mm in diameter) were excised from leaves of 2-week-old tomato plants, placed on water agar in petri dishes, and then sprayed with a spore suspension of GHA (1 × 10^9^ spores/mL, ca. 0.1 mL per leaf disc) and with tomato powdery mildew *P. neolycopersici* (approximately 1 × 10^4^ spores/mL). The dishes were then placed in a growth chamber for 10 days, and, then, total DNA was extracted from the leaf discs using the NucleoMag Plant kit (MACHEREY-NAGEL). Fungal biomass in tomato leaves was quantified on the basis of the amplification of the *P. neolycopersici* alpha-tubulin gene using primer set (PnTub_F, TAATTCCTCGGGACTGCAAC; PnTub_R, CATCATCGGGTGAAGAAGGT) (Pathak et al., 2020) in 100 ng of total genomic DNA. Tomato ribulose-1,5-bisphosphate carboxylase/oxygenase (rubisco) gene was amplified using a primer set (Sl-rubisco_F, GAACAGTTTCTCACTGTTGAC; Sl-rubisco_R, CGTGAGAACCATAAGTCACC) (Mesarich et al., 2014) as a calibration standard. Quantitative real-time PCR analysis (qRT-PCR) was performed using the LightCycler 480 (Roche, Basel, Switzerland) with the KAPA SYBR Fast qPCR Kit (Nippon Genetics, Tokyo, Japan) according to the manufacturer’s instructions. Results were analyzed using the *E^−^* ^ΔCt^ method (Livak and Schmittgen, 2001) with an average of 11 biological replicates. Data were analyzed for significant differences using the Mann–Whitney *U*-test and R version 4.0.3.

## Results

### *B. bassiana* strain GHA-based bioinsecticide Botanigard suppressed severity of vegetable powdery mildews

**Figure 1:**
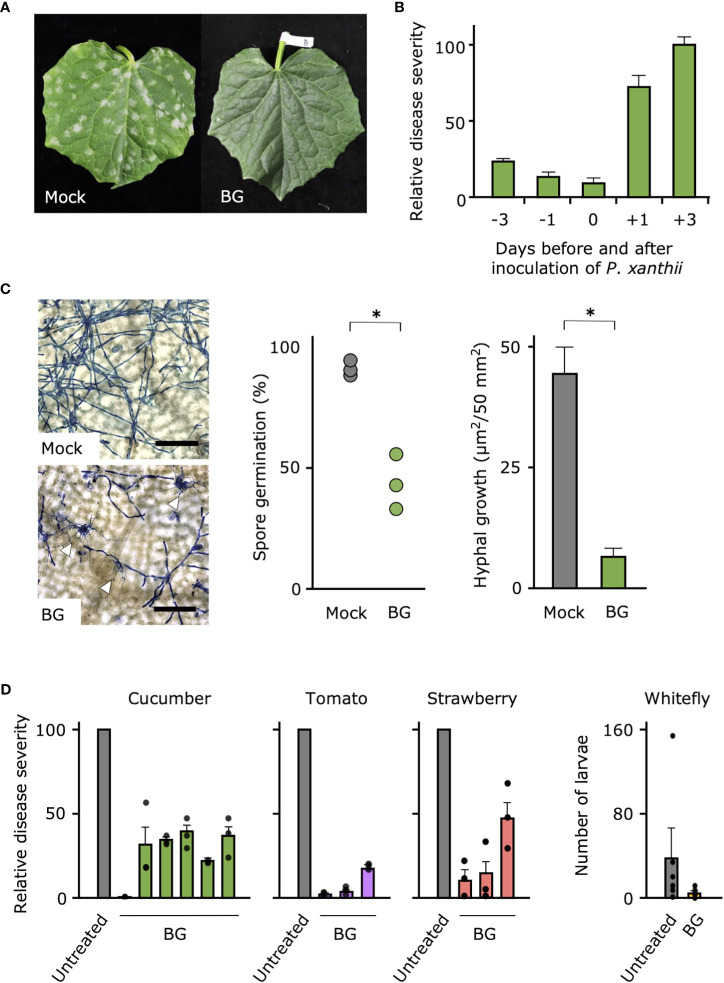
Biocontrol effect of bioinsecticide Botanigard against powdery mildews of vegetables and whitefly in the greenhouse. **(A)** Cucumber leaves were treated with distilled water (Mock) or a 1,000-fold dilution of Botanigard and, were inoculated with a spore suspension of powdery mildew *P. xanthii*. Photograph was taken 10 days after inoculation. **(B)** Mean relative disease severity (± SD) on the cucumber leaves that were treated with a 1,000-fold dilution of Botanigard from 3 days before to 3 days after inoculation with a spore suspension of *P. xanthii*. Disease severity was assessed 12 days after inoculation with mean disease severity on mock plants set as 100. **(C)** Spore germination and hyphal growth on leaves stained with lactophenol aniline blue were measured using a microscope. Hyphae of *P. xanthii* are thick, whereas those of *B. bassiana* are thin. Arrow heads indicate hyphal interaction. Bars = 100 µm. Asterisks indicate a significant difference compared to the mock treatment (*p < 0.01) in the Mann–Whitney *U*-test. **(D)** Cucumber, tomato, and strawberry plants in the greenhouse were treated with 1,000-fold dilution of Botanigard or not (Untreated). The mean disease severity on the mock plants was set as 100, and the relative disease severity on treated plants was determined. Field trials were replicated at different locations as described in **Supplemental Table 1** (*N* = 6 for cucumbers and *N* = 3 for tomatoes and strawberries, three replications in each trial). The whitefly larvae and adults were counted and means were compared between treatments.

Because only the 500- and 1,000-fold dilutions of Botanigard were suppressive against cucumber powdery mildew, for further tests, we used the 1,000-fold dilution (ca. 1 × 10^7^ GHA spores/mL), the concentration generally used to control insect pests (**Supplemental Figure 1**). When we tested the efficacy of the bioinsecticide against cucumber powdery mildew in the growth chamber, Botanigard applied to the cucumber leaves before inoculation with *P. xanthii* completely suppressed symptoms in contrast to the controls, which had typical symptoms with slight yellowing by 10 days after inoculation (**Figure 1A**; **Supplemental Figure 2A**). In our comparison of disease severities on the cucumber leaves treated with Botanigard at different times before or after inoculation with *P. xanthii*, symptom development was reduced by 75%–90% only when plants were pretreated 1 or 3 days before inoculation with *P. xanthii*, indicating the prophylactic effect (**Figure 1B**). Pretreatment with Botanigard also had reduced spore germination of *P. xanthii* by about half compared with the untreated control (**Figure 1C**). The hyphal network of *P. xanthii* covered the leaf surface by 3 days after inoculation, but the hyphal growth was reduced about 75% by application of Botanigard. The thick hyphae of *P. xanthii* rarely overlapped with the thinner hyphae of *B. bassiana* (**Supplemental Figure 2B**).

In the greenhouse and field trials of the efficacy of Botanigard against powdery mildews of five vegetables (cucumber, melon, tomato, eggplant, and strawberry) (**Supplemental Table 1**), disease severity after natural infection or inoculation with the respective powdery mildews was suppressed between 50% and 100% by pretreatment of Botanigard (**Figure 1D**; **Supplemental Table 2**). As expected, this bioinsecticide reduced the number of whitefly larvae and adults on tomato plants in the greenhouse (**Figure 1D**), indicating that it can control the insect pests and powdery mildews simultaneously.

### *B. bassiana* strain GHA suppressed severity of cucumber powdery mildew and induced HR-like cell death

**Figure 2:**
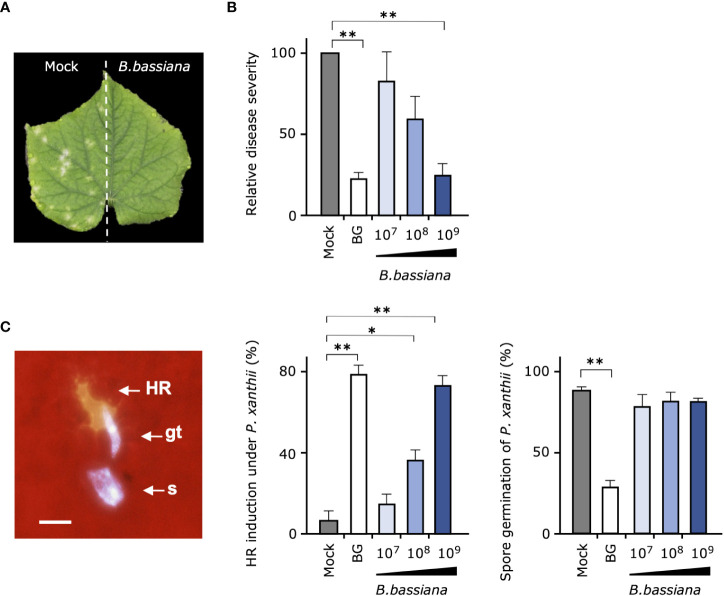
Suppressive effect of cucumber powdery mildew and induction of plant resistance by *Beauveria bassiana* strain GHA. **(A)** One-half of each cucumber leaf was treated with a spore suspension of *B. bassiana* GHA (1 × 10^9^ spores/mL) and the other half with water (Mock), and, then, the entire leaf was inoculated with a spore suspension of powdery mildew *Podosphaera xanthii*. A representative leaf at 10 days after inoculation is shown. **(B, C)** Leaves of cucumber treated with distilled water (Mock), 1,000-fold dilution of Botanigard or a spore suspension of *B. bassiana* (1 × 10^7^, 1 × 10^8^, or 1 × 10^9^ spores/mL) and then inoculated with a spore suspension of *P. xanthii*. **(B)** Relative disease severity at 12 days after inoculation based on average severity on mock-treated plants as 100. **(C)** Hypersensitive response (HR)–like cell death in epidermal cells under germ tubes (gt) and spore (s), and spore germination of *P. xanthii* at 24 h. Bar = 20 µm. Asterisks indicate a significant difference compared to the mock treatment (*p < 0.005 and **p < 0.001) in the Mann–Whitney *U*-test.

We next investigated whether entomopathogenic fungus *B. bassiana* strain GHA, the active ingredient of Botanigard, is involved in the suppression of cucumber powdery mildew *P. xanthii*. First, to verify whether the biocontrol effect of strain GHA is systemic or not, one-half of the cucumber leaves grown in pots were treated with GHA and the another with water, and, then, the whole leaves were inoculated with *P. xanthii* spores. Typical symptoms appeared only on the mock-treated leaves, indicating that the biocontrol effect was limited to the area of GHA application (**Figure 2A**).

In the test to determine whether pretreatments of leaves with different concentrations of *B. bassiana* GHA induce resistance in cucumber, GHA had a concentration-dependent suppressive effect on powdery mildew (**Figure 2B**). In particular, pretreatment of a 1,000-fold dilution of Botanigard and high concentration of GHA (1 × 10^9^ spores/mL) showed a similar biocontrol effect against *P. xanthii*. When the leaf surfaces were observed with fluorescence microscopy at 24 h after inoculation, fluorescent epidermal cells were observed under germ tubes of *P. xanthii*, indicating a HR-like cell death (**Figure 2C**; **Supplemental Figure 3**). The pretreatments with either the bioinsecticide or GHA strain increased the number of HR-like cell death with increasing concentration of the active ingredient (**Figure 2C**). Spore germination of *P. xanthii* was inhibited about 60% by Botanigard, but not by GHA (**Figure 2C**). *B. bassiana* hyphae were rarely present around epidermal cells with HR-like cell death.

Cucumber roots were treated with spore suspension of GHA or distilled water and grown in pots for 2 weeks. The mass of aerial plant parts and roots did not differ significantly from the control, indicating that GHA is not a PGPF per se under this condition (**Supplemental Table 3**).

### Gene Ontology annotation of differentially expressed genes detected by RNA-seq analysis

**Figure 3:**
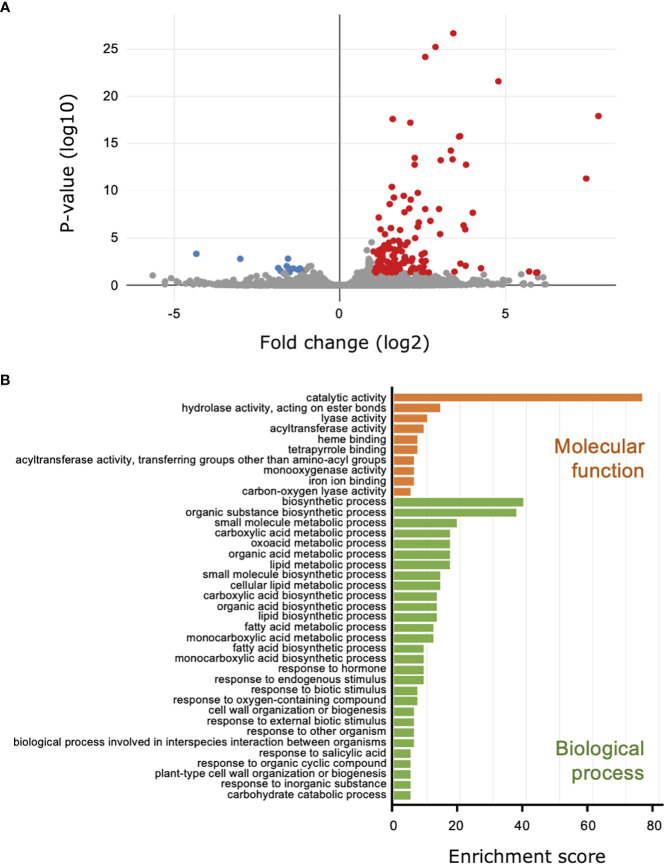
Transcriptome and Gene Ontology (GO) enrichment analysis of the cucumber leaves treated with *Beauveria bassiana* strain GHA. Leaves were treated with a spore suspension of GHA or mock and then inoculated with a spore suspension of powdery mildew *Podosphaera xanthii*. **(A)** Volcano plot of differentially expressed genes (DEGs) between GHA- and mock-treated cucumber leaves. Red and blue points represent upregulated and downregulated DEGs, respectively (*p* < 0.05). **(B)** GO enrichment analysis of upregulated DEGs. GO enrichment were classified by GO terms for molecular function (orange) and biological process (green) (*p* < 0.05).

**Figure 4:**
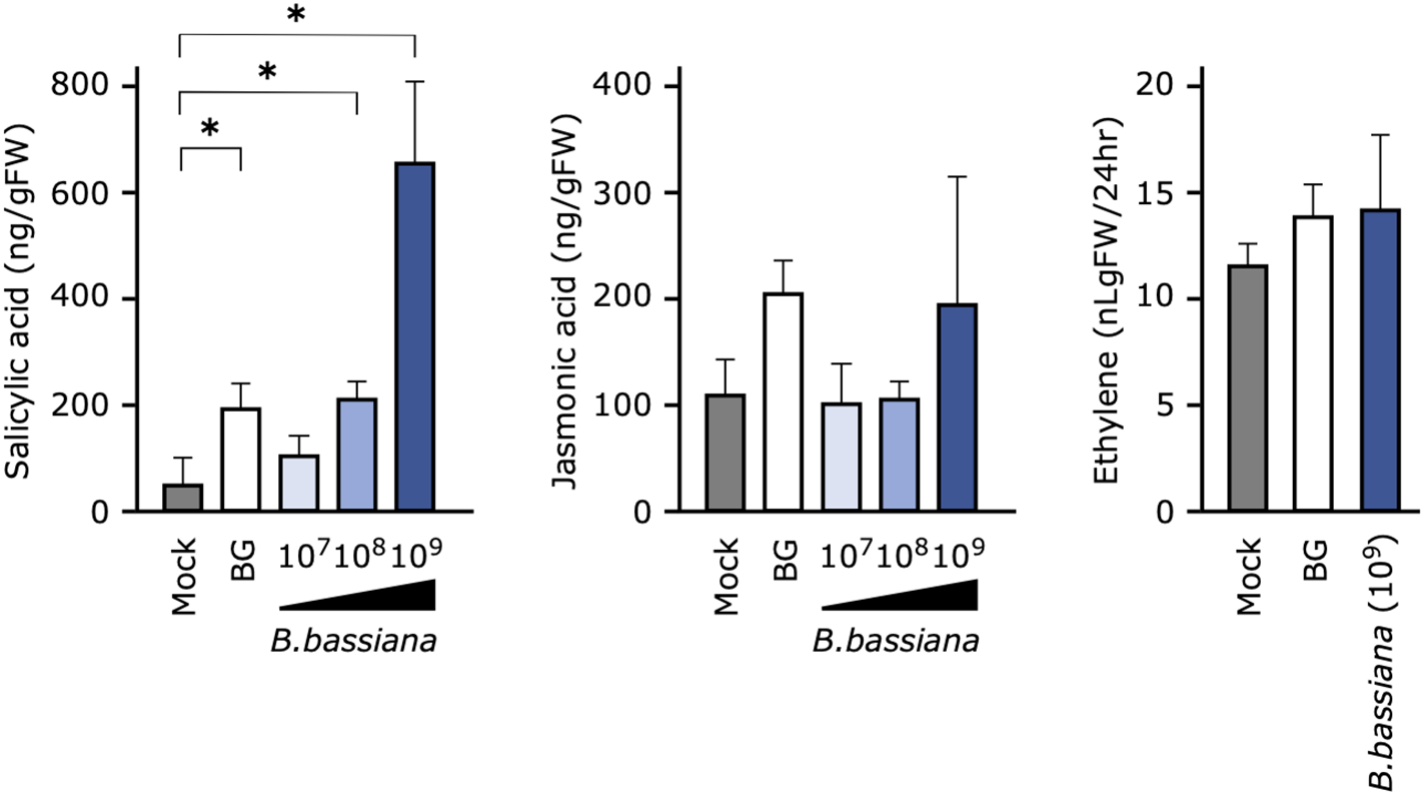
Effects of pretreatment of cucumber seedlings with *Beauveria bassiana* GHA on phytohormone levels at 24 h after inoculation. Seedlings were sprayed with distilled water (Mock), a 1,000-fold dilution of Botaniguard or a spore suspension of *B. bassiana* (1 × 10^7^, 1 × 10^8^, or 1 × 10^9^ spores/mL), and inoculated with a spore suspension of powdery mildew *Podosphaera xanthii*. Data are means ± SD of three independent measurements. Asterisks indicate a significant difference compared to the mock treatment at *p* < 0.05 in the Mann–Whitney *U*-test.

For the comparison of the transcriptomes from the cucumber leaves to investigate the influence of *B. bassiana* colonization on the plant, 26.7 million reads were mapped to the cucumber genome, and 150 DEGs were identified; 137 DEGs were upregulated and 13 were downregulated (**Figure 3A**; **Supplemental Table 4**; **Figure 4**). We did not have enough transcripts for *P. xanthii* and GHA to map to the fungal genomes.

In the GO annotations with a cutoff of *p* < 0.05, the 137 upregulated DEGs were classified into 39 functional subgroups, including 10 in molecular function and 29 in cellular component (**Figure 3B**). For molecular function, the GO terms heme binding (GO:0020037), tetrapyrrole binding (GO:0046906), and iron ion binding (GO:0005506), which all may be related to iron uptake, were detected. For cellular component, the DEGs were significantly enriched for the primary metabolic pathway and biosynthetic processes for carboxylic acid, oxoacid, organic acids, lipids, and fatty acids (**Figure 3B**; **Supplemental Table 5**). DEGs that were upregulated during the response of cucumber plants to *B. bassiana* GHA and powdery mildew were enriched for response to biotic stimulus (GO:0009607), response to external biotic stimulus (GO:0043207), response to other organism (GO:0051707), and biological process involved in interspecies interaction between organisms (GO:0044419) (**Figure 3B**). Two GO terms (GO:0071554, cell wall organization or biogenesis; and GO:0071669, plant-type cell wall organization or biogenesis) indicated reconstitution of plant cell walls in response to powdery mildew (**Figure 3B**). In addition, DEGs were significantly enriched in two GOs related to phytohormones including SA, which is involved in induction of plant resistance (GO:0009725 and GO:0009751).

### *B. bassiana* GHA induced accumulation of SA, but not JA and ET

To determine whether the phytohormone is involved in *B. bassiana* GHA-mediated resistance to cucumber powdery mildew, a quantitative analysis of endogenous phytohormones was performed. SA accumulated significantly in leaves treated with Botanigard or with a high concentration of GHA (1 × 10^8^ and 1 × 10^9^ spores/mL), but the levels of JA and ET did not change significantly (**Figure 4**).

### Biocontrol effect of *B. bassiana* GHA on NahG tomatoes against tomato powdery mildew

**Figure 5:**
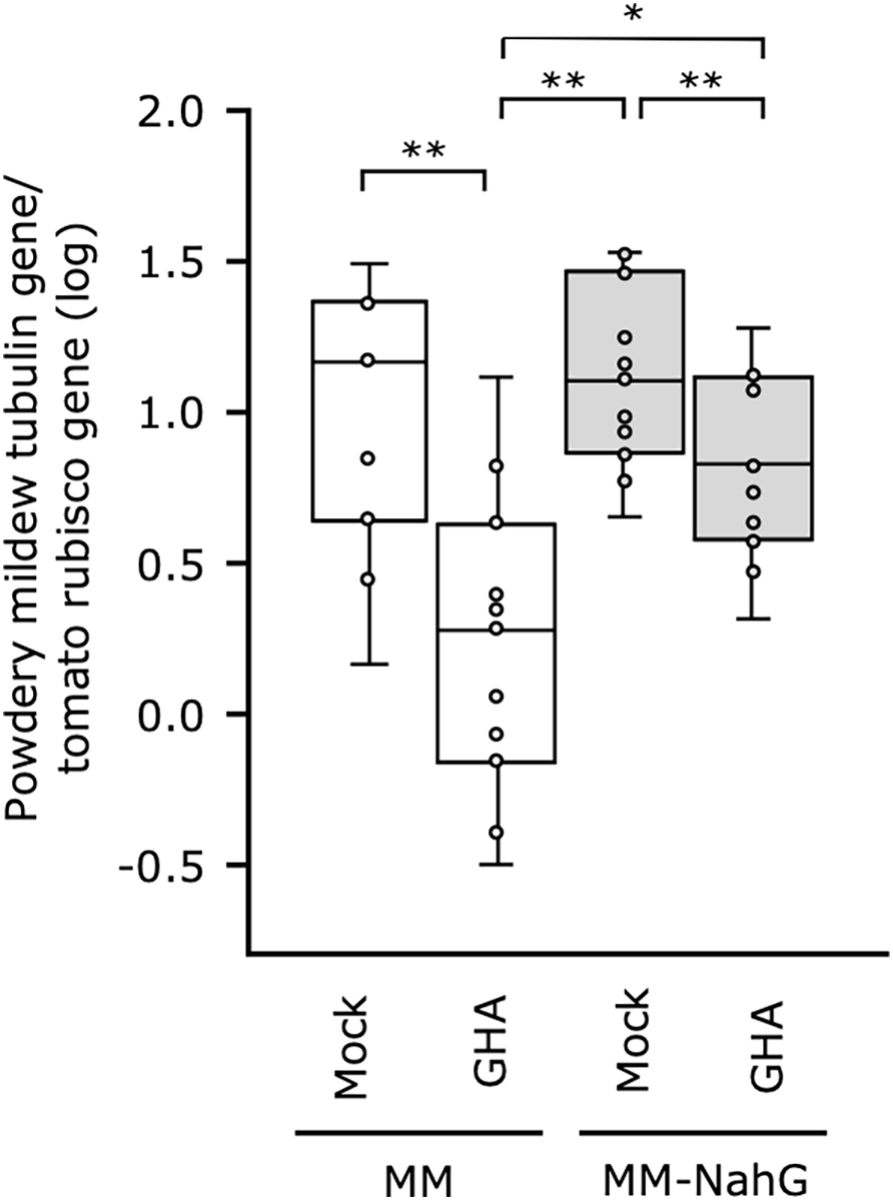
Effect of *Beauveria bassiana* GHA on NahG tomato plants against tomato powdery mildew. Leaf disks of cv. Moneymaker (MM) and Moneymaker-NahG (MM-NahG) were sprayed with distilled water (Mock) or a spore suspension of *B. bassiana* (1 × 10^9^ spores/mL) and then inoculated with tomato powdery mildew *Pseudoidium neolycopersici*. Fungal biomass in tomato leaves was quantified by a quantitative real-time PCR on the basis of amplification of the alpha-tubulin gene of *P. neolycopersici* compared with the ribulose-1,5-bisphosphate carboxylase/oxygenase (rubisco) gene in tomato. Asterisks indicate a significant difference (*p < 0.05 and **p < 0.005) in the Mann–Whitney *U*-test.

To further elucidate the influence of SA in *B. bassiana* GHA-mediated resistance, we established a tomato system with MM-NahG, which is unable to accumulate SA, and powdery mildew *P. neolycopersici*. As NahG plant responds excessively to inoculation with tomato powdery mildew, leaf discs were prepared and sprayed with the spore suspension of *P. neolycopersici*. Powdery mildew symptoms appeared on all leaves including the mock control and did not visibly differ in severity (**Supplemental Figure 5**), so fungal biomass in the tomato leaves was quantified by qRT-PCR. The GHA treatment of the MM tomato did inhibit powdery mildew growth compared with the untreated control but was not as effective in suppressing growth in the MM-NahG (**Figure 5**). A significant difference in fungal biomass was also found for MM and MM-NahG treated with GHA. These results suggest that SA is partially involved in the resistance induced after treatment with *B. bassiana* GHA.

## Discussion

Because *B. bassiana* was isolated as an antagonist of wheat take*-*all fungus *Gaeumannomyces graminis* (Renwick et al., 1991), its application for disease control has been attempting. Some strains are effective against root and basal rot of common onion caused by *Fusarium oxysporum* f. sp. *cepae* and damping-off caused by *Rhizoctonia solani* (Flori and Roberti, 1993; Ownley et al., 2008; Bamisile et al., 2018). However, the modes of action for disease control are still unclear. Here, endophytic strain GHA induced HR-like cell death against powdery mildew *P. xanthii*, leading to a significant decrease in disease symptoms. The biocontrol effect of GHA was local, but hyphae of *P. xanthii* and *B. bassiana* did not always overlap on the leaf surface. These findings suggest that GHA prevents the penetration of *P. xanthii* indirectly by stimulating plant resistance.

Although the transcriptome analysis with RNA-seq revealed that gene expression in cucumber plants was not altered substantially, 137 genes were upregulated and 13 were downregulated. Among the GO terms annotated for upregulated DEGs, three GO terms in molecular function were associated with the regulation of iron uptake, which plays a critical role in the generation of reactive oxygen intermediates during immunity. Iron catalyzes hydrogen peroxide to generate more damaging reactive oxygen species, causing intracellular damage and, ultimately, programmed cell death (Herlihy et al., 2020). On the other hand, iron deficiency activates phytohormones that are used for plant immune signaling, suggesting cross-talk between iron and plant immunity (Herlihy et al., 2020; García-Espinoza et al., 2023). A GO term associated with SA, which activates plant immunity to biotrophic and hemibiotrophic pathogens such as powdery mildews (Zhang and Li, 2019), was also detected, including genes encoding a WRKY transcription factor (*CsWRKY59*) and three BTB/POZ and TAZ proteins. WRKYs act as central regulators of complex networks in many aspects of plant immunity (Pieterse et al., 2012; Wani et al., 2021). The expression of *CsWRKY59* was positively regulated against *P. xanthii* in this study but is negatively regulated in response to inoculation with other powdery mildew *Podosphaera fusca* (Chen et al., 2020b). The role of the protein–protein interaction motifs BTB/POZ and TAZ in plant immunity is not well defined. In *A. thaliana*, the BTB/POZ domain of NPR1, a key regulator of SA-dependent systemic resistance, activates the pathogenesis-related (PR) gene *PR-1* by derepressing its function through binding to the transcriptional repressor TGA2 (Boyle et al., 2009). In fact, only SA accumulated in the GHA-treated cucumber leaves in the present study, but the JA and ET levels did not increase at the same time point. Moreover, the biocontrol effect of GHA on NahG tomato line, which is deficient in SA accumulation, was partially reduced against powdery mildew infection. Endophytic strains FRh2 and BG11, which induce SAR in *A. thaliana* to the plant pathogen *S. sclerotiorum*, differed in which genes involved in phytoalexin, JA and SA signaling pathways, and glucosinolates they induced; however, accumulation of JA, SA, or glucosinolates were not significantly altered (Raad et al., 2019). Only the BG11 strain acted as a PGPF and increased root biomass. Together, the biocontrol activity and the mode of action of *B. bassiana* differ considerably depending on the strain.

Against cucumber powdery mildew, *B. bassiana* GHA isolated from the bioinsecticide induced plant resistance but did not inhibit spore germination of *P. xanthii* as the bioinsecticide did, suggesting that other components in Botanigard could be involved in the control of spore germination. Some of the petroleum distillates used in Botanigard are also used as agricultural spray oils and fungicides. For example, a machine oil is a spiracle-blocking insecticide and a fungicide that inhibits spore germination and development of powdery mildews including *P. xanthii* (Ohtsuka and Nakazawa, 1991; Ohtsuka et al., 1991). The oil components in Botanigard may thus inhibit spore germination of *P. xanthii*. Spores that did germinate could then be prevented from penetrating by SA-mediated local resistance induced by the GHA strain, assuming that the fungus and the oil components in this bioinsecticide act in a coordinated manner.

Maize plants treated with endophytic *B. bassiana* reduce feeding damage by the european corn borer *Ostrinia nubilalis*, but the infection rate of this insect by *B. bassiana* is low (Bing and Lewis, 1991). Thus, a secondary metabolite produced by endophytic *B. bassiana* in the plant is suspected to be involved in feeding inhibition (Bing and Lewis, 1991; Wagner and Lewis, 2000). GHA has not yet been shown to produce secondary metabolites in plants that could be responsible for the suppressive effect of the powdery mildews, and no genes related to the biosynthesis of secondary metabolites were detected in the transcriptome data.

The symbiotic association established between entomopathogenic fungi and plants as endophytes and PGPFs is progressively being revealed to be of great importance in nature. *B. bassiana* induces proteins related to photosynthesis and energy metabolism, which could enhance plant growth (Gómez-Vidal et al., 2009; Raad et al., 2019). Endophytic entomopathogens *B. bassiana* and *M. robertsii* provide plants with nitrogen from insect carcasses (Behie et al., 2012; Behie and Bidochka, 2014). Furthermore, in *M. robertsii*, nutrient exchange is bidirectional; it acquires carbon from the plant that is converted to trehalose and chitin, a fungal cell wall component (Behie et al., 2017). The capacity of entomopathogenic fungi to translocate nitrogen from insect to plants could help reduce the use of chemical fertilizers. *B. bassiana* GHA endophytically colonizes on tomato and cucumber plants (Nishi et al., 2020) but could not provide cucumber plants with nitrogen from mealworm larvae (data not shown). Because plant growth did not differ between GHA-treated plants and the untreated controls, GHA does not promote plant growth either. Thus, GHA might be specific for protecting plants from insect pests and pathogens.

Entomopathogenic fungi are known to have a potential to control plant diseases and insect pests as endophytes, epiphytes, and PGPFs in various plants (Jaber and Ownley, 2018). In this study, the bioinsecticide Botanigard, with endophytic *B. bassiana* GHA (Nishi et al., 2020) as an active ingredient, strongly suppressed the occurrence of powdery mildews (*P. xanthii*, *P. neolycopersici*, *P. aphanis*, and *S. fuliginea*) of vegetable plants at the dilution commonly used to control insect pests, indicating the dual control of insect pests and pathogens is possible. Thus, Botanigard was officially approved in 2019 for use as a biological insecticide/fungicide in Japan. The GHA strain is able to penetrate a scratched plant epidermis and to colonize within the plant tissue and can be reisolated at high frequency, indicating that it is an endophytic strain (Nishi et al., 2020). In general, the epidermis of vegetable plants is easily damaged during management operations such as bud picking, leaf removing, and supporting them on poles. Therefore, applying Botanigard after these operations may improve the colonization rate of GHA in plant tissues. In the future, *B. bassiana* GHA will be developed as a more versatile biopesticide if its range of application against other important plant pathogens and insect pests is determined.

## Data availability statement

The original contributions presented in the study are publicly available. This data can be found here: https://www.ncbi.nlm.nih.gov/bioproject/PRJDB15569.

## Ethics statement

The manuscript presents research on animals that do not require ethical approval for their study.

## Author contributions

YI designed the study with the support of SY, KKu, and MKu. YI and MKu managed the research funding 29008B and 02028C, respectively. KY, SA, TK, KN, KKa, CT, AS, NY, MF, FT, and MKu collected field and greenhouse data with the support of SM. YH, ON, MKo, KM, and YI collected laboratory data. YI and YH did the RNA-sequencing and analyzed and annotated the data. YI, MK, and KM did the mass spectrometry analyses. YI wrote the paper. All authors contributed to the article and approved the submitted version.
