# Supplementary material for: Entomopathogenic fungus Beauveria bassiana–based bioinsecticide suppresses severity of powdery mildews of vegetables by inducing the plant defense responses
Source: Front Plant Sci. 2023 Aug 24;14:1211825. doi: 10.3389/fpls.2023.1211825 (PMC10484095; doi:10.3389/fpls.2023.1211825)
Supplement: Supplementary file 1 [file DataSheet_1.pdf]

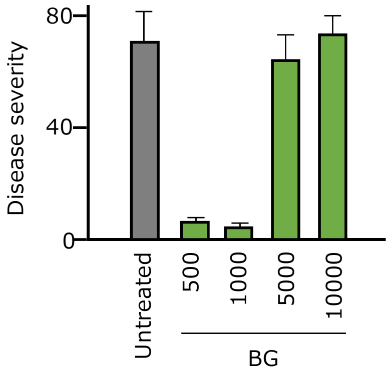

Supplemental figure 1. Suppressive effect of Botanigard ES at different concentrations against cucumber powdery mildew, *Podosphaera xanthi*.
Cucumber plants were sprayed with a 500 to 10000-fold diluted Botaniguard ES (BG) and inoculated with a spore suspension of *P. xanthii*. Disease severity was assessed 12 days after inoculation with mean disease severity on mock plants set as 100 (3 replications).

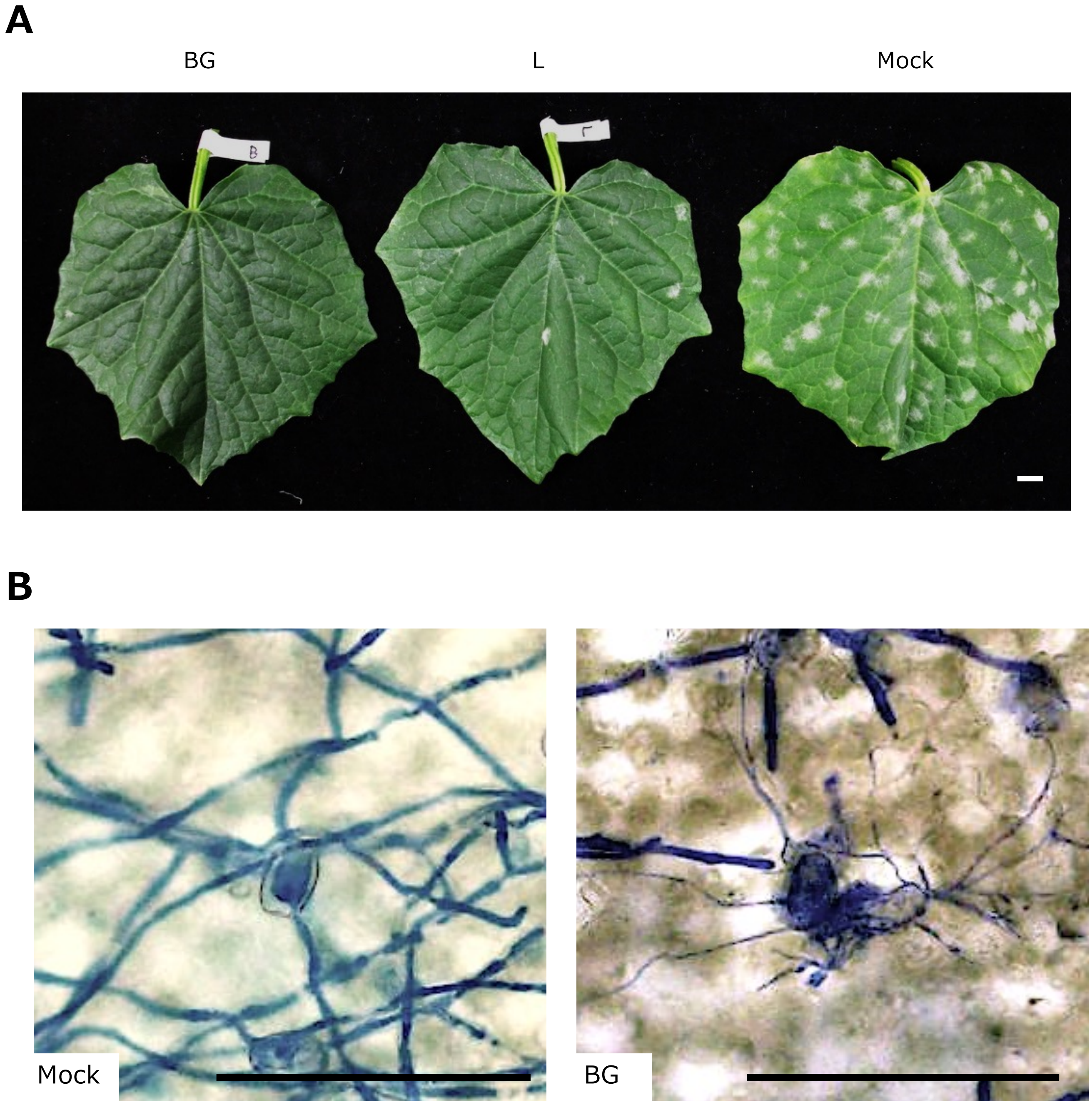

Supplemental figure 2. Disease symptom and hyphal development of powdery mildew Podosphaera xanthii on the cucumber leaves treated with Botanigard.
(A) Powdery mildew symptoms shown in Fig. 1A. Cucumber leaves were treated with a 1000-fold dilution of Botanigard (BG), *Lecanicillium*-based bioinsecticide (L) (data not shown), or distilled water (Mock), and 5 min later, were inoculated with a spore suspension of powdery mildew *P. xanthii*. Bar represents 1 cm. (B) Stained hypha on leaves shown in Fig. 1C. Hyphae of *P. xanthii* are thick, those of *B. bassiana* are thin. Bars represent 100 μm.

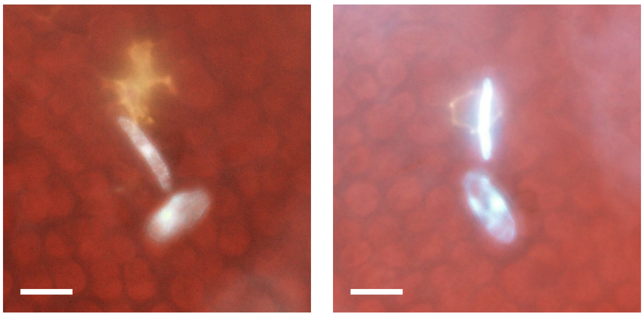

Supplemental figure 3. Suppressive effect on spore germination of cucumber powdery mildew, *Podosphaera xanthi*, and induction of hypersensitive response (HR) in cucumber by *Beauveria bassiana* strain GHA.
Cucumber plants were sprayed with a spore suspension of GHA, and then with a spore suspension of *Podosphaera xanthii*. Photographs were taken 24 hours post inoculation. HR-like cell death was observed under germ tubes of *P. xanthii* spores. Bar represents 20 μm.

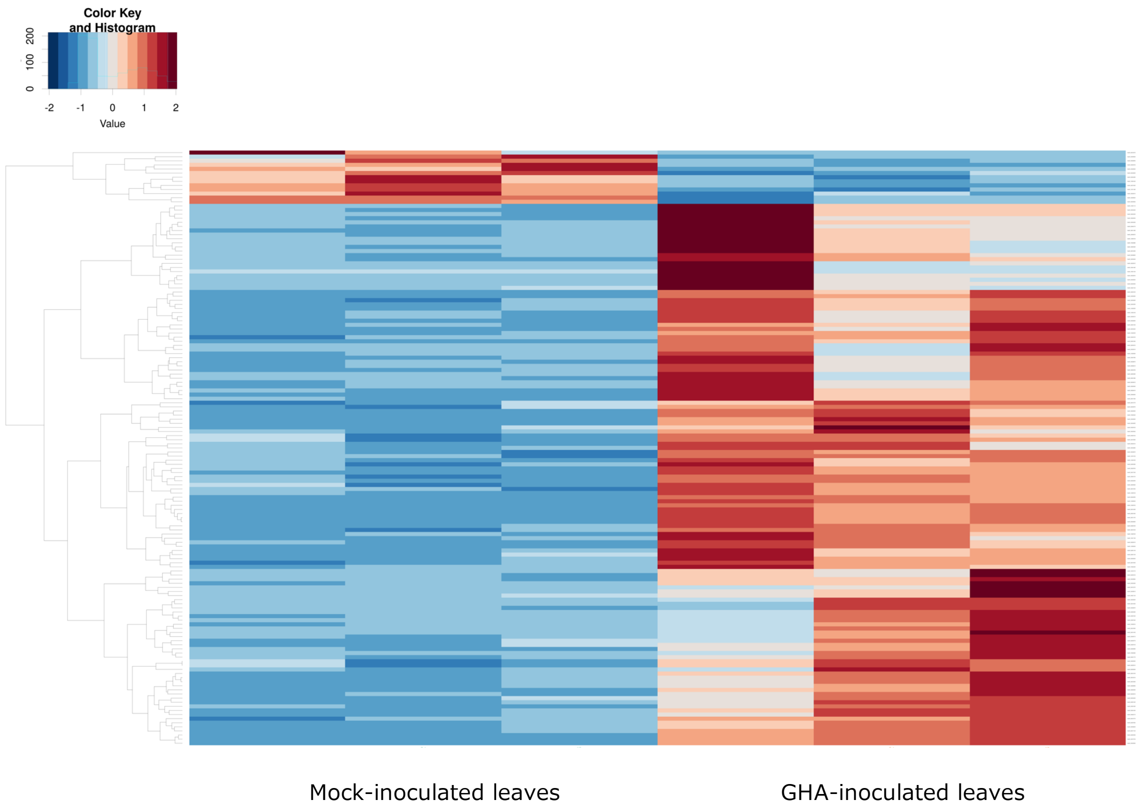

Supplemental figure 4. Heat map of differentially expressed genes (DEGs) in cucumber leaves inoculated with Beauveria bassiana strain GHA.
Cucumber plants were sprayed with a spore suspension of GHA or water (Mock), and then inoculated with a spore suspension of powdery mildew *Podosphaera xanthii*. DEGs were determined using DESeq2 based on *p*-value < 0.05 adjusted by the Benjamini and Hochberg method and log2|fold change| > 1. The list of DEGs is available in supplemental table 4.

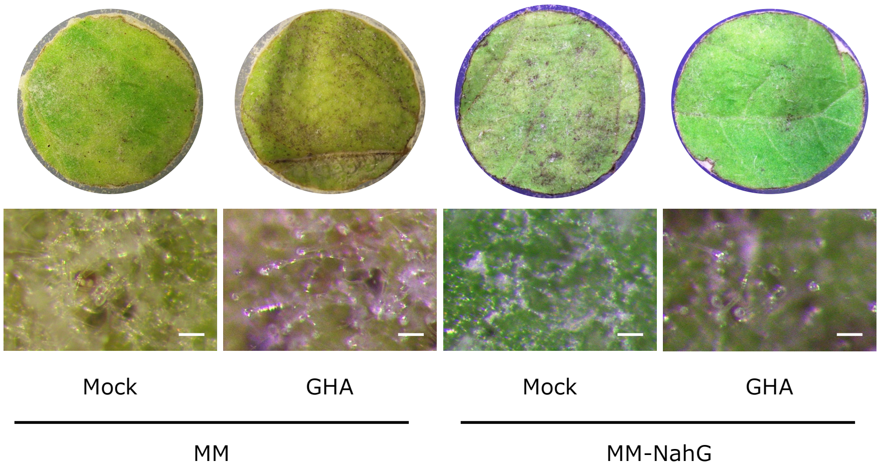

Supplemental figure 5. Disease symptoms and sporulation of powdery mildew *Pseudoidium neolycopersici* on tomato lines treated with *Beauveria bassiana* GHA.
Leaf disks of cv. Moneymaker (MM) and Moneymaker-NahG (MM-NahG) were sprayed with distilled water (Mock) or a spore suspension of *B. bassiana* GHA (1×10^9 spores/mL), then inoculated with tomato powdery mildew *P. neolycopersici*. Symptoms on leaf disks (upper row) were assessed and sporulation (lower row) was observed under a stereomicroscope 10 days after inoculation.
